# The use of ^18^F-FDG PET to differentiate progressive disease from treatment induced necrosis in high grade glioma

**DOI:** 10.1007/s11060-015-1883-1

**Published:** 2015-09-18

**Authors:** J. W. Dankbaar, T. J. Snijders, P. A. Robe, T. Seute, W. Eppinga, J. Hendrikse, B. De Keizer

**Affiliations:** Department of Radiology and Nuclear Medicine, University Medical Center Utrecht, PO Box 85500, 3508 GA Utrecht, The Netherlands; Department of Neurology and Neurosurgery, Brain Center Rudolf Magnus, University Medical Center Utrecht, Utrecht, The Netherlands; Department of Radiotherapy, University Medical Center Utrecht, Utrecht, The Netherlands

**Keywords:** High grade glioma, FDG PET, MRI, Progressive disease, Treatment induced necrosis

## Abstract

In the follow-up of patients treated for high grade glioma, differentiation between progressive disease (PD) and treatment-induced necrosis (TIN) is challenging. The purpose of this study is to evaluate the diagnostic accuracy of FDG PET for the differentiation between TIN and PD after high grade glioma treatment. We retrospectively identified patients between January 2011 and July 2013 that met the following criteria: age >18; glioma grade 3 or 4; treatment with radiotherapy or chemoradiotherapy; new or progressive enhancement on post treatment MRI; FDG PET within 4 weeks of MRI. Absolute and relative (to contralateral white matter) values of SUVmax and SUVpeak were determined in new enhancing lesions on MRI. The outcome of PD or TIN was determined by neurosurgical biopsy/resection, follow-up MRI, or clinical deterioration. The association between FDG PET and outcome was analyzed with univariate logistic regression and ROC analysis for: all lesions, lesions >10, >15, and >20 mm. We included 30 patients (5 grade 3 and 25 grade 4), with 39 enhancing lesions on MRI. Twenty-nine lesions represented PD and 10 TIN. Absolute and relative values of SUVmax and SUVpeak showed no significant differences between PD and TIN. ROC analysis showed highest AUCs for relative SUVpeak in all lesion sizes. Relative SUVpeak for lesions >20 mm showed reasonable discriminative properties [AUC 0.69 (0.41–0.96)]. FDG PET has reasonable discriminative properties for differentiation of PD from TIN in high grade gliomas larger than 20 mm. Overall diagnostic performance is insufficient to guide clinical decision-making.

## Introduction

High grade gliomas are the most common malignant primary brain tumors, with glioblastoma multiforme (GBM) accounting for 15 % of intracranial neoplasms. Fewer than 10 % of GBM patients survive beyond a period of 5 years [1]. Treatment of GBM typically involves neurosurgery followed by radiotherapy with concomitant and adjuvant chemotherapy [2]. In anaplastic gliomas (WHO grade 3), most patients will receive radiotherapy, some with concomitant and adjuvant chemotherapy (temozolomide) or post-radiotherapy chemotherapy (procarbazine-CCNU-vincristine, PCV) [3]. In the follow-up of these patients, it is essential to rapidly and accurately identify recurrent or progressive tumor (progressive disease, PD). In neuro-oncological imaging, this has proven to be a continuing challenge due to overlapping imaging characteristics of PD and treatment-induced necrosis (TIN), also referred to as pseudoprogression [4]. TIN causes new or increasing contrast-enhancing lesion(s) on MRI within the original high-dose radiation field. This strongly resembles the radiological aspect of PD [5, 6]. TIN may be identified by the spontaneous stabilization or regression of the contrast-enhancing lesion over time, requiring follow-up imaging. This delays the diagnosis of PD causing delayed insight in the ineffectiveness of chemotherapy, and thereby unnecessary continuation of treatment.

The most reliable method to confirm PD is tissue analysis after neurosurgical biopsy or resection. However, each neurosurgical intervention carries the risks associated with neurosurgery. In addition, false negative results may occur due to sampling errors, especially in biopsies. As a result the diagnosis is often based on non-invasive methods [7].

^18^F-FDG (FDG) PET has shown to be able to accurately identify areas of active disease in brain tumors [8]. In addition FDG uptake correlates with tumor grade and aggressiveness [8, 9]. However, the use of FDG PET for the differentiation between TIN and PD has remained limited. The total number of case studies using FDG PET for this purpose is small. In general, lesions that are suspicious for PD on MR imaging (MRI) that show increased FDG uptake are likely to represent PD. When using FDG PET for this purpose, low sensitivity is an important problem. Small foci of PD may be hard to identify. In recent years FDG PET imaging has shown great improvement. The spatial resolution has increased dramatically and the possibility to co-register the FDG PET images to the MRI has improved the diagnostic performances of FDG PET in other fields of medicine [10–12].

The purpose of this study is to evaluate the diagnostic accuracy of state-of-the art FDG PET for the differentiation between TIN and PD in patients with high grade glioma.

## Methods

### Study design and patient selection

In this retrospective cohort study we identified all patients treated for high grade glioma between January 2011 and July 2013 that met the following inclusion criteria: (1) age >18 years; (2) histologically proven WHO grade 3 or 4 glioma; (3) postoperative treatment with radiotherapy (RTX) with or without additional chemotherapy; (4) new or progressive enhancement on post treatment MRI; (5) FDG PET imaging within 4 weeks of the MRI. Data on age, sex, treatment type and time between RTX and MRI were collected.

The study was approved by the institutional review board.

### Treatment protocol

Patients were treated according to international guidelines.

All GBM patients with a good clinical condition (Karnofsky performance scale 70 or higher) and lack of contra-indication were treated with radiotherapy, concomitant chemotherapy (temozolomide: 75 mg/m^2^/day for 42 days), and adjuvant chemotherapy (temozolomide: 6 4-weekly adjuvant cycles: 200 mg/m^2^/day, 5 days on, 23 days off). Radiotherapy consisted of 30 fractions of 2 Gy, using intensity modulated radiotherapy (IMRT). Alternatively, GBM patients with high age and/or poor clinical condition, who were eligible for treatment, usually underwent a short schedule of radiotherapy (12 fractions of 3.5 Gy). Most patients with anaplastic glioma also underwent radiotherapy 30 × 2 Gy, without chemotherapy. After 2012, patients with anaplastic-oligodendroglial tumors and co-deletion of 1p and 19q underwent PCV chemotherapy after radiotherapy [13, 14].

### Post radiotherapy MRI protocol

In all high grade glioma patients at our institution MRI scans are performed within 4 weeks prior to radiotherapy. In GBM patients who undergo temozolomide-based chemoradiation, the first post-treatment MRI is obtained at 3–4 weeks after completion of the concomitant phase of chemoradiotherapy; in the patients receiving radiotherapy as monotherapy (both anaplastic gliomas and GBM), the first post-treatment MRI follows 12–16 weeks after radiotherapy. All MRIs were acquired at 1.5T (Philips Healthcare, Best, the Netherlands). The MRI protocol included FLAIR, T1, gadolinium enhanced 3D T1, and DWI sequences.

We identified cases of possible PD on the basis of increased or new enhancement on post-radiotherapy T1 gadolinium enhanced images within the high-dose radiation field.

### PET-protocol

A 10-min static acquisition of the brain was acquired on a PET/CT camera (Biograph TruePoint 40, Siemens Healthcare) starting 30 after intravenous injection of 2 MBq/kg 18F-FDG. All patients fasted for at least 6 h prior to imaging. A low-dose CT scan without contrast enhancement was used for attenuation correction (120 kV, 40 mAs). PET images were reconstructed using, TOF, point spread function and 4 iterations with 21 ordered subsets after attenuation correction.

### Imaging analysis (variables)

The initial MRI images were co-registered to the PET images. Regions of interest (ROI) were drawn in the area of new or increased enhancement shown on the MRI images and in the contralateral white matter. Measurement of SUVmax and SUVpeak were obtained in all ROIs. Relative values of SUVmax and SUVpeak were calculated as ratios between the values in the enhancing lesion and the SUVmean of contralateral white matter [15].

### Outcome

In this study, we included both patients with and without adjuvant temozolomide treatment after the initial FDG PET.

The presence of PD or TIN was determined by neurosurgical biopsy/resection, follow-up MRI, or clinical deterioration following the initial MRI.

On follow-up MRI, PD was defined according to RANO criteria as further increase of enhancing lesion(s) on contrast enhanced T1 images, adding up to an increase of more than 25 % in the sum of the products of perpendicular diameters compared to baseline (pre-radiotherapy) MRI [16, 17]. TIN was defined as any new enhancing lesion on contrast enhanced T1 that remained stable, decreased or disappeared after 12 weeks [17].

In cases where the patient underwent a second biopsy/resection of enhancing tissue, the final outcome is determined by the histological diagnosis (TIN or PD).

Finally, PD could also be diagnosed on the basis of clinical deterioration, if no follow-up imaging or tissue diagnosis was obtained. The diagnosis ‘clinical PD’ was based on the occurrence of clinical-neurological deterioration shortly (<12 weeks) after the MRI corresponding to the location of the lesion with increasing enhancement.

### Statistical analysis

The association between potential predictor variables and the outcome, PD, was analyzed by means of univariate logistic regression. Since the accuracy of FDG PET is greatly dependent on lesion size [18, 19], analyses were done separately for: (1) all lesions; (2) lesions larger than 10 mm on MRI; (3) lesions larger than 15 mm; (4) lesions larger than 20 mm. For each variable the odds ratio (OR) with the 95 % confidence interval (95 % CI) was determined. ROC curves were created for all variables. The AUC with 95 % CI of all ROC curves was determined. For the variable with the highest AUC the sensitivity, specificity, positive predictive value (PPV) and negative predictive value (NPV) were determined for the cutoff value with optimal predictive properties. Statistical computations were carried out with SPSS 19.0 (IBM Corporation, NY, USA).

## Results

Thirty-seven patients met the inclusion criteria. In 7 patients a final diagnosis of either PD or TIN could not be obtained. Three of these patients died shortly after the initial imaging. The other 4 patients were treated with additional anti-tumor therapy (mostly chemotherapy) after the initial imaging showed increased enhancement. Since further follow-up imaging showed stable or decreased enhancement, it was impossible to differentiate a positive effect of the treatment on PD from spontaneous regression of TIN in these 4 patients.

The characteristics of the remaining 30 patients are summarized in Table 1. Five patients had grade 3 glioma and 25 patients grade 4 glioma. Treatment consisted radiotherapy and temozolomide in 25 patients, radiotherapy only in 4 patients, and a combination of radiotherapy, carmustine (BCNU) and dibromodulcitol (as part of a clinical trial) in 1 patient. The median time between radiotherapy and the initial MRI was 10 months (range 3–101). The median time between this MRI and the FDG PET was 6 days (range −5 to 27 days). The 30 patients had a total of 39 enhancing lesions on MRI. 29 lesions represented PD and 10 TIN. The outcome was determined with MRI follow-up in 24 lesions, biopsy in 12 lesions and by the clinical course in 3 lesions. The average lesion diameter on MRI for PD was not significantly different from TIN (29.8 vs 30.2 mm, mean difference of 0.44 mm with a 95 %-CI of −14.3 to 13.8).

**Table 1:** Patients characteristics

Nr	Age	Type and grade	Treatment	MRI/RTX interval (months)	MRI lesion size (mm)	PET/MRI interval (days)	SUVmax	Relattive SUVmax	SUVpeak	Relative SUVpeak	Final diagnosis	Evidence
1	79	GBM 4	RT	5	25	6	6.8	3.3	4.8	2.4	PD	Clinical-FU
2	62	GBM 4	RT/TMZ	18	11	25	14.5	5.1	10.6	3.7	PD	MRI-FU
				18	15	25	7.8	2.7	5.4	1.9	PD	MRI-FU
				18	5	25	5.5	1.9	4.2	1.5	PD	MRI-FU
3	34	OA 3	RT	101	12	15	8.7	3.7	3.1	1.3	PD	Tissue
4	31	AA 3	RT	20	35	8	17.7	9.6	11.8	6.4	PD	MRI-FU
				20	6	8	21.6	11.7	7.6	4.1	PD	MRI-FU
5	56	GBM 4	RT/TMZ	11	21	4	8.5	3.1	3.8	1.4	PD	MRI-FU
6	47	GBM 4	RT/TMZ	8	57	7	6.7	3.0	5.9	2.7	PD	Tissue
7	61	GBM 4	RT/TMZ	7	26	−1	5.4	2.2	4.8	2.0	PD	Tissue
8	68	GBM 4	RT/TMZ	4	35	21	7.8	2.8	6.0	2.2	TIN	MRI-FU
9	55	GBM 4	RT/TMZ	42	9	6	7.6	3.2	5.1	2.1	PD	Tissue
10	59	GBM 4	RT/TMZ	21	10	7	9.5	4.8	6.5	3.3	TIN	MRI-FU
11	49	GBM 4	RT/TMZ	4	16	−5	5.8	2.6	4.7	2.1	TIN	Tissue
12	35	GBM 4	RT	4	29	6	19.2	10.2	12.9	6.9	PD	MRI-FU
13	48	AA 3	RT/BCNU/dibromo-dulcitol	20	9	27	7.7	3.6	4.7	2.2	TIN	MRI-FU
				20	22	27	6.7	3.1	4.5	2.1	TIN	MRI-FU
14	49	GBM 4	RT/TMZ	4	55	3	8.4	4.2	6.2	3.1	PD	Tissue
				4	56	3	10.4	4.4	7.6	3.2	PD	Tissue
15	53	GBM 4	RT/TMZ	10	14	2	8.2	3.8	6.2	2.9	PD	Clinical-FU
16	64	GBM 4	RT/TMZ	6	72	7	9.2	3.7	8.0	3.2	PD	Tissue
17	50	GBM 4	RT/TMZ	10	31	7	8.3	4.1	6.6	3.3	PD	Tissue
18	67	GBM 4	RT/TMZ	4	55	8	7.3	2.9	5.3	2.1	TIN	MRI-FU
19	59	GBM 4	RT/TMZ	7	34	5	8.9	3.1	7.4	2.6	PD	Tissue
20	56	GBM 4	RT/TMZ	8	17	7	13.7	4.4	11.6	3.7	PD	MRI-FU
21	41	AA 3	RT/TMZ	33	20	3	6.3	2.5	3.1	1.2	PD	MRI-FU
22	58	AA 3	RT/TMZ	13	47	10	7.3	2.7	5.2	2.1	PD	MRI-FU
				13	32	10	6.2	3.6	4.8	2.5	PD	MRI-FU
				13	17	10	6.2	2.7	4.5	1.9	PD	MRI-FU
23	57	GBM 4	RT/TMZ	15	29	4	14.6	4.7	11.9	3.8	PD	Tissue
24	63	GBM 4	RT/TMZ	3	60	6	8.5	3.7	6.6	2.9	PD	MRI-FU
25	66	GBM 4	RT/TMZ	12	64	15	10.4	4.6	8.6	3.8	PD	Clinical-FU
26	50	GBM 4	RT/TMZ	5	51	7	7.6	2.9	4.4	1.7	TIN	MRI-FU
				5	20	7	7.5	2.8	4.3	1.6	TIN	MRI-FU
27	51	GBM 4	RT/TMZ	23	14	3	3.8	1.9	2.1	1.0	PD	Tissue
28	56	GBM 4	RT/TMZ	12	48	5	8.9	3.4	5.5	2.1	TIN	MRI-FU
				24	36	13	22.2	9.8	14.9	6.6	TIN	MRI-FU
29	57	GBM 4	RT/TMZ	6	37	5	5.9	2.5	4.2	1.8	PD	Tissue
30	56	GBM 4	RT/TMZ	13	13	5	7.0	2.7	3.0	1.2	PD	MRI-FU

*Clinical-FU* clinical follow-up, *MRI-FU* follow-up MRI, *GBM* glioblastoma multiforme, *PD* progressive disease, *RT* radiotherapy, *TIN* treatment-induced necrosis, *Tissue* tissue diagnosis (biopsy or resection), *TMZ* temozolomide, *Relative SUVmax* SUVmax lesion/SUVmean normal contralateral white matter,* Relative SUVpeak* SUVpeak lesion/SUV mean normal contralateral white matter

**Fig. 1:**
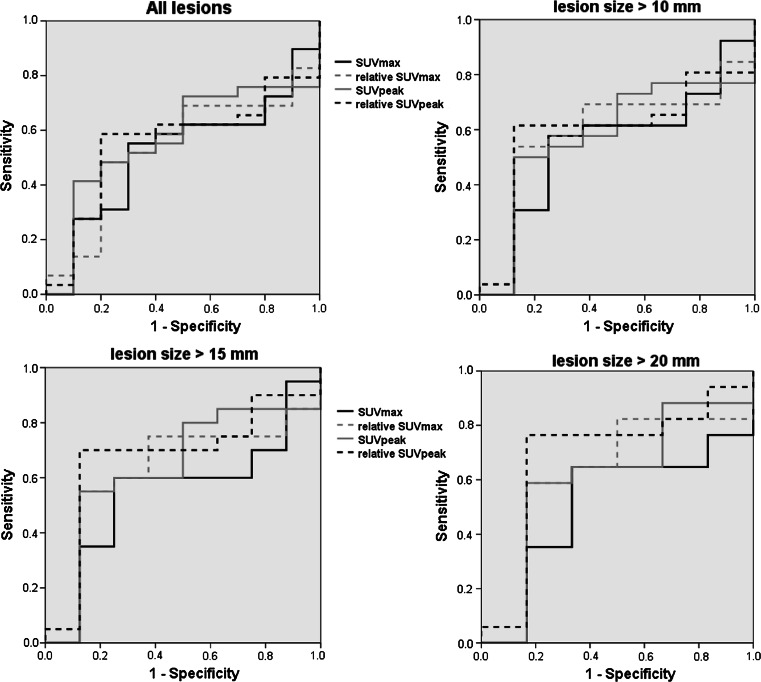
ROC curves by lesion size

The PET findings are summarized in Table 2. Absolute and relative values of SUVmax and SUVpeak showed no significant differences between PD and TIN. ROC analysis showed highest AUCs for relative SUVpeak in all lesion sizes (Fig. 1). The use of relative SUVpeak for lesion larger than 15 and 20 mm showed reasonable discriminative properties with AUCs of 0.68 (0.45–0.90) and 0.69 (0.41–0.96) respectively. For lesions larger than 20 mm a threshold value for the relative peak enhancement of 2.26 yielded a sensitivity of 0.76 (0.56–0.97), a specificity of 0.83 (0.54–1.13), a negative predictive value of 0.56 (0.23–0.88) and a positive predictive value of 0.93 (0.79–1.06) for PD.

**Table 2:** Discriminative properties of ^18^F-FDG PET for progressive disease and treatment induced necrosis

Lesions (PD/TIN)	PET parameter	PD	TIN	*p* value	OR (95 % CI)	AUC (95 % CI)
All (29/10)	SUVmax	9.4	9.1	0.85	1.02 (0.86–1.21)	0.52 (0.32–0.72)
	Relative SUVmax	4.1	3.9	0.72	1.05 (0.75–1.48)	0.54 (0.34–0.74)
	SUVpeak	6.5	6.1	0.73	1.05 (0.81–1.36)	0.57 (0.37–0.76)
	Relative SUVpeak	2.8	2.6	0.78	1.11 (0.64–1.95)	0.56 (0.36–0.75)
>10 mm (26/8)	SUVmax	9.2	9.2	0.97	1.00 (0.82–1.21)	0.55 (0.32–0.77)
	Relative SUVmax	3.9	3.8	0.86	1.04 (0.69–1.58)	0.60 (0.38–0.81)
	SUVpeak	6.6	6.2	0.78	1.04 (0.80–1.36)	0.58 (0.36–0.80)
	Relative SUVpeak	2.80	2.54	0.67	1.15 (0.62–2.11)	0.60 (0.38–0.81)
>15 mm (20/8)	SUVmax	9.43	9.23	0.92	1.01 (0.83–1.23)	0.55 (0.31–0.79)
	Relative SUVmax	4.12	3.78	0.72	1.09 (0.71–1.66)	0.63 (0.40–0.86)
	SUVpeak	7.00	6.19	0.54	1.10 (0.82–1.49)	0.64 (0.40–0.87)
	Relative SUVpeak	3.04	2.54	0.44	1.32 (0.66–2.64)	0.68 (0.45–0.90)
>20 mm (17/6)	SUVmax	9.55	10.09	0.81	0.97 (0.79–1.20)	0.51 (0.24–0.78)
	Relative SUVmax	4.28	4.14	0.90	1.03 (0.67–1.57)	0.63 (0.36–0.90)
	SUVpeak	7.11	6.75	0.81	1.04 (0.75–1.44)	0.61 (0.33–0.89)
	Relative SUVpeak	3.17	2.77	0.60	1.22 (0.60–2.50)	0.69 (0.41–0.96)

*PD* progressive disease, *TIN* treatment-induced necrosis, *OR* odds ratio, *AUC* area under the curve

**Fig. 2:**
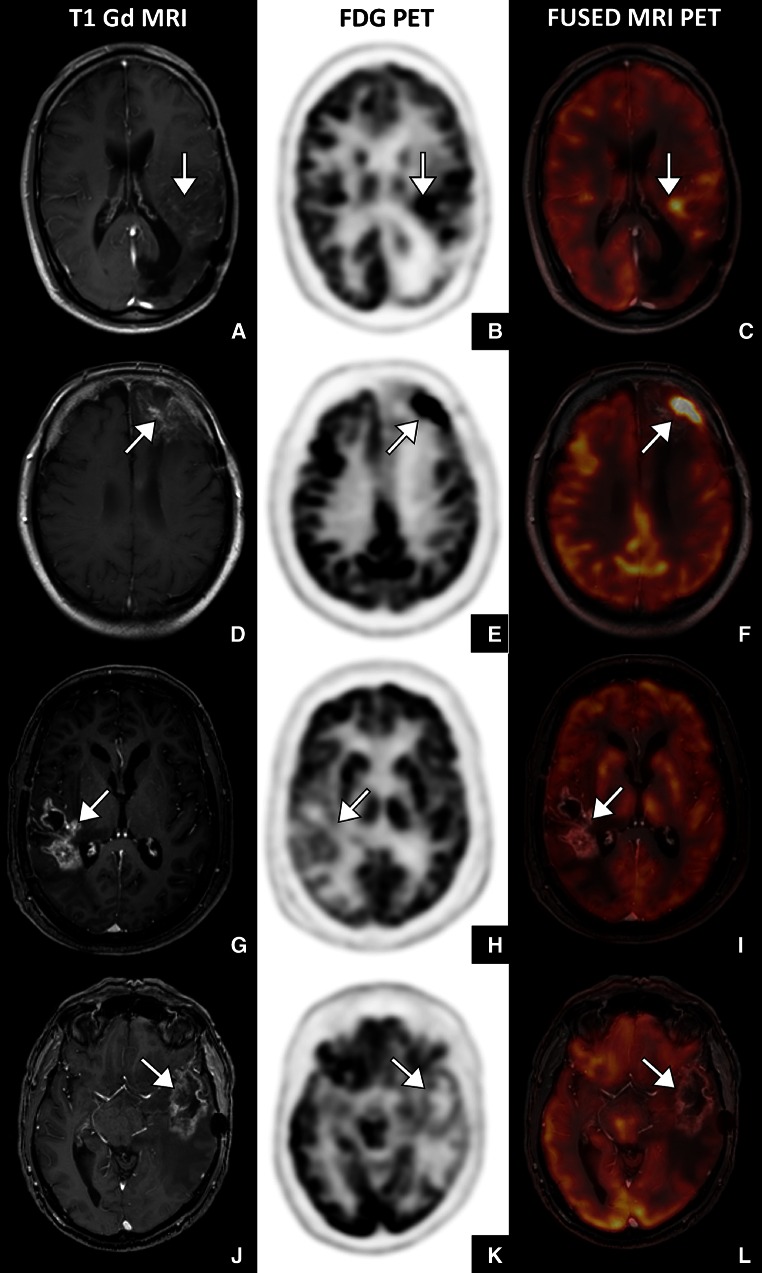
Four illustrative cases. **a–c** True positive FDG PET (patient 4) with an anaplastic astrocytoma presenting with a new enhancing lesion of 35 mm 20 months after radiotherapy. The lesion showed increased FDG uptake and proved to represent progressive disease (PD) on follow-up MRI. **d–f** False positive FDG PET (patient 28) with glioblastoma multiforme (GBM) presenting with a new enhancing lesion of 48 mm 12 months after chemoradiotherapy. Increased FDG uptake was seen laterally in the lesion. The lesion proved to represent treatment induced necrosis (TIN) on follow-up MRI. The increased FDG uptake may have been caused by status epilepticus. **g–i** False negative FDG PET (patient 6) with GBM presenting with a new enhancing lesion of 57 mm 8 months after chemoradiotherapy. The lesion did not show increased FDG uptake. Tissue analysis after neurosurgical biopsy showed PD. **j–l** True negative FDG PET (patient 18) with GBM presenting with a new enhancing lesion of 55 mm 4 months after chemoradiotherapy. The lesion did not show increased FDG uptake and proved to represent TIN on follow-up MRI

Figure 2 shows illustrative cases with true positive, false positive, true negative, and false positive FDG PET findings.

## Discussion

Our results show reasonable discriminative properties of FDG PET for lesions larger than 20 mm. However, adding smaller lesion clearly makes FDG PET for the differentiation between PD and TIN unreliable. The differentiation between PD and TIN with FDG PET therefore remains difficult.

A recent meta-analysis of the discriminative properties of FDG PET for PD in high-grade glioma patients showed sensitivities ranging between 0.18 and 1.00 with a summarized sensitivity of 0.79 (95 % CI 0.67–0.88) [20]. Specificities ranged between 0.25 and 1.00 with a summarized specificity of 0.70 (95 % CI 0.50–0.84) [20]. These values are similar to our findings. The maximum number of patients in the studies selected for the meta-analysis was 44 with an average of 22 patients [21], making our series relatively large. In the meta-analysis only few studies used treatment strategies that are consistent with the currently used treatment protocols (i.e. temozolomide-based multimodality therapy) [20]. In our study 81 % of the patients received a combination of radiotherapy and temozolomide.

The use of FDG PET for the differentiation of PD from TIN is hampered by several features. Firstly conditions like status epilepticus increase glucose metabolism in parts of the brain, thereby mimicking tumor activity (Fig. 2g–i, patient 6). Secondly, glucose metabolism may be increased by radiation induced inflammation, resulting in false-positive findings for PD. Thirdly, despite continuing improvement of spatial resolution PET imaging still has low sensitivity for smaller lesions. This is also illustrated by our results that show increasing sensitivity with increasing lesion size. The co-registration of the PET images with MRI has significantly improved lesion detection. However, our results show that FDG PET seems to be insufficient to differentiate between PD and TIN in small lesions. Further improvement of the resolution can be expected with the development of combined PET/MR systems and new detectors.

To improve the detection of tumor with PET other tracers are being investigated. Since progressing tumors exhibit increased amino acid transport, amino acid analogs, such as O-2-^18^F-fluoroethyl-l-tyrosine (^18^F–FET); 3,4-dihydroxy-6–^18^F-fluoro-l-phenylalanine (^18^F-FDOPA); and L-methyl-^11^C-methionine (^11^C-MET) seem to be interesting candidates for the differentiation between TIN and PD [7, 22]. These tracers show lower uptake in normal cortex making more accurate detection possible. A recent meta-analysis evaluating the use of ^11^C-MET PET for the detection of glioma recurrence showed high sensitivity (0.87) and specificity (0.81) [21].

MRI-based imaging techniques, such as perfusion weighted imaging [23–26], diffusion weighted imaging [27] and MR spectroscopy [26, 28], and other imaging techniques like SPECT [29], and dynamic contrast enhanced CT [30] also have reasonable discriminative properties for PD and TIN. However, none of the techniques are sufficiently reliable to completely guide clinical decision-making in all cases. Thereby multimodal imaging may be needed to provide stronger diagnostic information [4, 7]. Combined PET/MRI-scanning, with different PET tracers as well as combination of advanced MRI-techniques with FDG may prove useful and should be further studied in the future. However, mixed lesions containing both TIN and PD will most likely remain challenging for any combination of imaging techniques.

Due to several limitations our result need to be interpreted with caution. Firstly, we included only 30 patients with 39 lesions in our analysis. In the analysis with lesions larger than 20 mm only 23 lesions were included of which only 6 lesions proved to be TIN. Secondly, the use of follow-up imaging as a gold standard for PD is difficult. High grade glioma has a very high progression rate exceeding 90 % within 5 years in GBM. This implies that the longer you follow the patient, the more likely it is that the patient will develop PD. This progression may however not have been present at the time of the diagnostic dilemma of differentiating TIN from PD. The only true gold standard would be a representative (i.e. sufficiently large) tissue specimen, taken exactly from the enhancing area on MRI that was suspicious of PD, at the time of first presentation. This poses an ethical problem, since neurosurgical biopsy carries an important risk of complications. In our study only one-third of the patients underwent neurosurgical biopsy. Thirdly, the time between FDG PET and MRI was relatively long in some patients. Again this may have resulted in PD having occurred after the initial imaging. Fourthly, the retrospective study design resulted in the exclusion of several patients. We excluded all patients (n = 4) that were suspected of having PD based on the FDG PET images and therefore received additional treatment. The inhibiting effect of the treatment on further tumor growth could not be differentiated from non-progressive TIN already present at the start of the additional treatment. A prospective analysis is however difficult since patients that are suspected to have PD ought to receive additional treatment. Fifthly, selection bias has most likely occurred. FDG PET is not a standard procedure in the follow-up of high grade glioma patients in our institution. Only cases with hard to interpret MRI findings are likely to receive FDG PET. Although the effect of this selection on the results is difficult to assess, the selection bias may have contributed to the limited value of FDG PET in our study population. However, this selection forms a good representation of the population of interest, since advanced imaging for differentiation of TIN from PD is most relevant in patients with ambiguous findings on conventional MR imaging.

In conclusion, we evaluated the diagnostic properties of advanced FDG PET imaging with MRI-coregistration for the differentiation between TIN from PD in the follow-up of irradiated high-grade gliomas. Our data confirm the results from previous studies, showing that FDG PET provides some degree of differentiation between TIN and PD. However, in small lesions (<20 mm diameter) the diagnostic performance of FDG PET is poor and in larger lesions the performance is only reasonable. This is most likely not sufficient to fully guide clinical decision-making.
